# Ethical Pragmatism in Healthcare Management: A Multidimensional Framework for Responsible Decision-Making Under Real-World Constraints

**DOI:** 10.7759/cureus.117569

**Published:** 2026-10-06

**Authors:** Ahmad Abujaber, Abdulqadir J Nashwan

**Affiliations:** 1 Nursing, Hamad Medical Corporation, Doha, QAT; 2 Nursing and Midwifery Research, Certified Global Nurse Consultant, Doha, QAT

**Keywords:** ethical pragmatism, executive decision-making, healthcare ethics, healthcare governance, healthcare leadership, healthcare management, health policy, organizational ethics, organizational sustainability

## Abstract

Healthcare leaders frequently make decisions under conditions of uncertainty, competing stakeholder interests, resource constraints, and changing clinical and organizational demands. Established ethical traditions provide normative guidance, while procedural approaches such as Accountability for Reasonableness emphasize transparency, relevance of reasons, opportunities for revision and appeal, and mechanisms for ensuring fair healthcare decision-making. However, executive decisions are also shaped by organizational realities, societal and institutional conditions, and consequences that may unfold across different time horizons. This paper develops a healthcare-management-specific conceptualization of Ethical Pragmatism and proposes the Ethical Pragmatism Decision Landscape (EPDL) as a multidimensional representation of executive deliberation across four interacting domains: ethical foundations, organizational realities, societal and institutional context, and temporal perspective. Whereas Accountability for Reasonableness primarily addresses the legitimacy and procedural fairness of priority-setting decisions, the EPDL adds a structured approach for examining how ethical principles, operational feasibility, contextual pressures, stakeholder interests, and short- and long-term consequences interact in the formation and evaluation of executive decisions. Within the EPDL, Ethical Elevation is introduced as a comparative deliberative concept for examining the ethical defensibility of available options. The Most Ethically Sustainable Decision (MESD) represents the option judged most ethically defensible and practically sustainable among those reasonably available within a defined context. The framework is illustrated through applications to resource allocation, workforce planning, emergency department overcrowding, public health emergencies, strategic decision-making, governance, and health policy. Ethical Pragmatism is intended to complement rather than replace established ethical and procedural approaches by extending transparent justification and revisability into a broader multidimensional process of executive ethical deliberation. Further conceptual and empirical research is needed to examine the framework’s applicability, clarify its constructs, and evaluate its potential value in healthcare executive decision-making.

## Introduction and background

Healthcare organizations can be understood as complex systems in which clinical, technological, organizational, and social influences interact under conditions that are not entirely predictable [1-2]. Within these settings, executive decisions may involve several relevant ethical commitments alongside organizational conditions that shape available courses of action and their foreseeable consequences [2]. Decisions concerning workforce allocation, emergency department overcrowding, critical care capacity, service prioritization, and resource distribution may affect patients, healthcare professionals, organizations, and communities differently, requiring leaders to interpret competing claims within the circumstances in which action must occur.

Healthcare management involves ethical decision-making within complex organizational settings in which clinical responsibilities, organizational conditions, and wider societal considerations may intersect. Established ethical principles and professional obligations provide important normative reference points for healthcare leadership, yet their interpretation and application may become challenging when decisions involve competing priorities, uncertainty, and resource constraints [1, 3].

The complexity of these decisions reflects, in part, the diversity of ethical thought. Moral philosophy comprises several established traditions that direct attention to different aspects of ethical reasoning. Virtue ethics emphasizes character and practical wisdom [2], deontology grounds moral judgment in duties and principles derived from reason [3], and utilitarianism evaluates actions primarily in relation to their consequences [4]. Ross's theory of prima facie duties recognizes that legitimate moral obligations may conflict and require contextual judgment [5]. Subsequent contributions, including Rawls' conception of justice as fairness [6], Gilligan's care perspective [7], and principlism [8], further broaden the perspectives available for ethical analysis.

The expression "ethical pragmatism" has also been used in different ways within philosophical scholarship. Richardson developed constructive ethical pragmatism as an approach to practical moral reasoning concerned with the articulation and revision of norms within a moral community [9, 10]. Donelson employed the expression in a different argument, examining whether practical moral deliberation must depend on claims concerning objective moral truth or broader metaethical commitments [11]. These accounts demonstrate that ethical pragmatism does not refer to a single established framework and provide relevant points of comparison for the present inquiry. However, neither was developed specifically as a framework for healthcare executive decision-making.

Existing accounts of ethical pragmatism provide important perspectives on practical moral reasoning but do not directly address the particular conditions of healthcare executive decision-making examined in this paper. Healthcare leaders may be required to interpret ethical and professional obligations alongside workforce availability, financial and operational capacity, legal requirements, societal expectations, and consequences that unfold across different time horizons. The issue is not whether ethical commitments should yield to organizational pressure, but how those commitments can be interpreted and pursued when available options are constrained. Their consequences extend across different groups and over time.

Against this background, this paper develops a healthcare-management-specific conceptualization of Ethical Pragmatism. It introduces the Ethical Pragmatism Decision Landscape (EPDL) as a framework for examining executive deliberation across ethical foundations, organizational realities, societal and institutional context, and temporal perspective. It further introduces Ethical Elevation and the Most Ethically Sustainable Decision (MESD) as concepts for comparing available courses of action within this landscape. The framework is intended to complement established ethical approaches while providing a basis for further conceptual refinement and empirical investigation.

## Review

Philosophical foundations for ethical pragmatism

Ethics is often presented as a body of knowledge that guides human conduct. In healthcare, ethical decision-making is frequently discussed through principles such as beneficence, non-maleficence, justice, and respect for autonomy [12]. While these principles provide an important foundation for ethical deliberation, the development of moral philosophy reveals a plurality of traditions that differ in how they understand moral judgment, obligation, and responsibility. Examining these traditions is therefore important for understanding the different philosophical foundations that may inform ethical reasoning in healthcare.

One of the earliest and most influential traditions is Aristotle's virtue ethics, which emphasizes the character of the moral agent rather than focusing solely on individual actions. Aristotle associated ethical judgment with the cultivation of virtue and the exercise of practical wisdom (phronesis) in particular circumstances [2]. In contrast, Kantian deontology grounds moral obligation in principles of reason and duty. Kant's categorical imperative requires the moral agent to act only on a maxim that could at the same time be willed as a universal law [3]. Utilitarian thinkers, including Bentham and later Mill, place greater emphasis on the consequences of action. Bentham's principle of utility evaluates actions according to their tendency to increase or diminish happiness [13]. At the same time, Mill holds that actions are right insofar as they tend to promote happiness and wrong insofar as they tend toward its opposite [4].

Subsequent developments further broadened ethical inquiry. Ross introduced the concept of prima facie duties to describe multiple morally significant obligations that may conflict, with actual duty depending on the circumstances of the particular case [5]. Rawls developed justice as fairness as a political conception of justice concerned with basic liberties, fair equality of opportunity, and the distribution of social and economic advantages [6]. Gilligan challenged dominant accounts of moral development by highlighting a different moral voice in which relationships, responsibility, care, and responsiveness to others assume greater significance [7]. Within biomedical ethics, Beauchamp and Childress organized ethical analysis around four principles: respect for autonomy, nonmaleficence, beneficence, and justice, which may require specification and balancing when applied to particular cases [8].

Philosophical pragmatism provides a further point of reference by directing attention to experience, inquiry, and the practical implications of ideas. James presented pragmatism as a method for examining the practical differences associated with competing ideas [14]. At the same time, Dewey situated moral conduct within lived experience and the social conditions in which action occurs [15].

Subsequent scholarship has developed different accounts explicitly associated with ethical pragmatism. Richardson developed constructive ethical pragmatism around the articulation and revision of moral norms through practical reasoning, including circumstances in which moral considerations are not fully determinate [9, 10]. Donelson used the term for a different philosophical argument concerning whether practical moral deliberation needs to depend on claims about moral truth or broader metaethical commitments [11]. These accounts differ substantially in purpose, but both illustrate attempts to understand moral deliberation without reducing it to the mechanical application of a predetermined rule.

These traditions do not provide identical accounts of moral responsibility. They variously emphasize moral character and practical wisdom, duties and obligations, consequences, justice, relationships and care, ethical principles, experience and practical consequences, and the deliberation required when moral considerations conflict. Their differences suggest that complex ethical questions may be examined through more than one form of moral reasoning, with different perspectives drawing attention to different features of a decision. The framework developed in this paper builds on this philosophical plurality while retaining the normative importance of established ethical principles, professional obligations, and standards of accountability.

Conceptual Propositions

Proposition 1: Established ethical traditions direct attention to different aspects of moral judgment, including character and practical wisdom, duties, consequences, justice, relationships and care, and ethical principles.

Proposition 2: Complex healthcare decisions may be examined through multiple ethical perspectives, each of which may draw attention to different morally relevant features of the decision.

Ethics as a multidimensional concept

The diversity of ethical traditions outlined in the previous section provides a basis for considering ethical reasoning as multidimensional rather than as the application of a single moral principle. Individual ethical theories offer coherent approaches to moral judgment. Still, they differ in the questions they prioritize, the considerations they regard as morally significant, and the criteria they use to evaluate action. In complex situations, several of these considerations may be relevant simultaneously, even when they point toward different conclusions. Ethical analysis may therefore involve examining a decision through multiple perspectives rather than assuming that one normative lens necessarily captures all of its morally relevant dimensions [2-11].

The significance of this plurality becomes more apparent when the same situation is examined through different ethical perspectives. For example, allocating a single intensive care bed under conditions of scarcity may raise different considerations depending on the ethical perspective applied. Utilitarian reasoning may emphasize the consequences of allocation and the overall benefit that can be achieved; justice-based reasoning may focus on the fairness of the allocation process and the distribution of scarce resources. At the same time, an ethic of care may draw greater attention to relationships, responsibilities, and responsiveness to those affected. The same decision may therefore raise several morally relevant considerations, whose prominence differs depending on the ethical perspective through which the situation is examined [4,6-8].

In healthcare organizations, ethical reasoning also takes place within organizational and societal contexts. Decisions concerning workforce planning, service prioritization, budgeting, technology adoption, and emergency preparedness may simultaneously involve considerations related to patient welfare, professional obligations, legal and regulatory requirements, resource availability, financial sustainability, public accountability, and longer-term organizational capacity. These considerations are not necessarily equivalent in ethical status, but they may interact to shape the options available to decision-makers. Their interaction occurs within environments characterized by uncertainty, resource constraints, and changing clinical, organizational, and policy conditions [1,8,16].

A multidimensional account of ethical reasoning should not be understood as moral relativism or as a rejection of established ethical theories. Ethical principles and professional obligations continue to provide normative reference points and boundaries for action, while applicable legal and regulatory requirements impose additional requirements on decision-making [8,16].

Multidimensionality instead concerns how different morally relevant considerations are examined when they point toward different priorities and how ethical commitments are interpreted and applied under particular contextual conditions. This perspective provides a basis for representing healthcare executive decision-making as navigation through interacting ethical and contextual considerations rather than as movement along a single continuum between ethics and pragmatism [1,8,16].

Conceptual Propositions

Proposition 3: Healthcare executive decisions may involve multiple morally relevant considerations whose relative significance depends partly on the features of the decision and its context.

Proposition 4: Healthcare executive decision-making may be conceptualized multidimensionally when ethical considerations interact with organizational and broader contextual conditions.

Relevance of ethical pragmatism to healthcare management

Healthcare executives make decisions in settings where clinical needs, organizational capacity, legal and regulatory requirements, public expectations, and resource limitations frequently converge. Choices concerning staffing, service configuration, patient flow, investment, and emergency response can affect patients, professionals, organizations, and communities in different ways. Ethical considerations are therefore embedded in many aspects of executive decision-making, including decisions that may initially appear primarily operational, financial, or strategic [1,8].

The complexity described earlier has practical consequences for executive deliberation. Interventions may interact with existing organizational conditions in ways that produce effects beyond those initially anticipated. At the same time, leaders may need to act before information is complete or future outcomes are clear. Executive decisions therefore cannot always be treated as isolated problems with stable conditions and fixed alternatives [1].

Within these conditions, healthcare decisions may involve several ethically relevant considerations at the same time. Expanding access may strain workforce capacity; measures intended to protect workforce wellbeing under staffing constraints may affect short-term service capacity; and concentrating limited resources in one area may reduce their availability elsewhere. During a public health emergency, ethical responsibilities toward individual patients may coexist with responsibilities toward the wider population. At the same time, decisions are also constrained by the capacity required to maintain essential health services. Available options may therefore preserve some ethical commitments more effectively than others [1,8,16].

Nonideal approaches in bioethics offer a useful point of comparison because they direct attention to moral reasoning under existing, rather than idealized, social and institutional conditions [16]. Applied to healthcare management, this perspective draws attention to conditions such as workforce availability, existing organizational structures, resource constraints, and distributions of influence that shape the context in which ethical responsibilities are exercised. These conditions do not by themselves determine what is ethically acceptable, but they may affect which courses of action are feasible and the consequences associated with them [16].

Established ethical theories remain essential because they provide principles, obligations, values, and standards through which decisions can be ethically examined. Their application in healthcare management may nevertheless require interpretation as circumstances, available information, and operational conditions change. Ethical deliberation can therefore be understood as potentially iterative rather than confined to a single decision point: an initial judgment may require reassessment as new information becomes available, conditions change, or previously uncertain consequences become apparent [2-8].

In this paper, pragmatism does not mean expediency, convenience, or subordinating ethical commitments to organizational performance. Rather, it refers to sustained attention to how ethical commitments can be translated into action within the conditions in which decisions must be made and implemented. Feasibility does not independently establish the ethical acceptability of a decision, but it influences whether and how an ethically supported course of action can be implemented. A feasible option may remain ethically unacceptable, while an ethically preferable option may require adaptation to prevailing conditions before it can be implemented safely or sustained over time [9-11,16].

The Ethical Pragmatism Decision Landscape (EPDL) is proposed as a conceptual representation of this form of deliberation. It conceptualizes healthcare executive decision-making as navigation among interacting ethical and contextual considerations rather than as the mechanical application of a single ethical doctrine or as a simple trade-off between ethical commitments and practical feasibility.

Conceptual Propositions

Proposition 5: Context-sensitive ethical judgment may be particularly important when healthcare decisions involve uncertainty, interdependence, and resource constraints.

Proposition 6: Organizational conditions may influence the feasibility and consequences of ethically supported courses of action but do not independently determine their ethical acceptability.

Proposition 7: Ethical deliberation in healthcare executive decision-making may be conceptualized as an iterative process in which ethical commitments are considered alongside changing information, contextual conditions, feasible options, and emerging consequences.

Defining ethical pragmatism

The expression ethical pragmatism has been used in different philosophical contexts and therefore requires clarification. Richardson developed constructive ethical pragmatism, an approach in which conceptions of the right and the good remain open to revision in light of one another, particularly when moral deliberation involves conflicting or incommensurable considerations [9]. His later account further examines how indeterminacies in morality may be addressed through the specification and introduction of moral norms [10]. Donelson uses ethical pragmatism in a different sense, arguing that moral deliberation can proceed without reference to moral truths or, more broadly, to metaethics [11]. The present paper does not adopt either account directly; rather, it develops a healthcare-management-specific use of the term while retaining established ethical theories and professional obligations as normative reference points.

In this paper, ethical pragmatism refers specifically to healthcare management. Established ethical traditions and professional obligations remain normative reference points, while legal, regulatory, organizational, and other contextual conditions shape how those commitments must be interpreted and pursued. The pragmatic element focuses on what can be implemented, with what resources, by whom, and with what reasonably foreseeable consequences.

Ethical pragmatism is a healthcare-management conceptual framework for examining how executive leaders interpret and pursue ethical commitments within organizational, societal, and institutional conditions, while considering how the consequences of available courses of action may unfold across different time horizons.

This definition establishes several boundaries. First, ethical pragmatism is not proposed as a new normative ethical theory. It does not replace established approaches such as virtue ethics, deontology, consequentialism, care ethics, theories of justice, or principlism. Second, it does not equate practicality or feasibility with ethical acceptability. Operational convenience, financial advantage, or institutional preference cannot independently justify a course of action. Third, it does not assume that all ethical considerations or stakeholder interests carry equal weight in every situation. Decisions remain subject to relevant ethical and professional obligations, applicable legal and regulatory requirements, reasoned justification, and appropriate accountability. The EPDL, developed in the following section, provides the proposed conceptual representation of this deliberative process.

Conceptual Propositions

Proposition 8: Ethical pragmatism, as defined here, is a healthcare-management conceptual framework rather than an independent normative ethical theory.

Proposition 9: Practical feasibility influences whether and how ethically supported courses of action can be implemented but is insufficient on its own to establish the ethical defensibility of a decision.

Proposition 10: Decisions examined through ethical pragmatism remain subject to relevant ethical and professional obligations, applicable legal and regulatory requirements, reasoned justification, and appropriate accountability.

The Ethical Pragmatism framework

The EPDL represents the proposed framework as a multidimensional model of healthcare executive deliberation. The landscape metaphor reflects the premise that ethically relevant and contextual considerations are not adequately represented by a single scale running from “ethical” to “pragmatic.” Instead, decision-makers navigate a changing terrain in which multiple courses of action may be available, each with different implications for ethical commitments, practical feasibility, affected stakeholders, and consequences across different time horizons.

Four domains organize the landscape. The first, ethical foundations, encompasses the normative considerations relevant to the decision. These may include beneficence, non-maleficence, justice, respect for autonomy, compassion, professional integrity, and stewardship, reflecting different ethical and professional traditions rather than a single normative framework [8]. The domain is not intended as an exhaustive list or a fixed hierarchy of values. The relevance and relative weight of particular considerations require reasoned justification in relation to the decision and its context.

The second domain, organizational realities, encompasses conditions that shape the feasibility and implementation of available courses of action. These may include workforce availability, financial resources, infrastructure, technological capability, and operational capacity. Such conditions can influence which options are practically available, how they can be implemented, and the consequences that may reasonably be anticipated [1]. Within the EPDL, organizational realities are therefore treated as contextual conditions within which ethical commitments must be translated into action, rather than as considerations that independently determine or override ethical obligations.

The third domain, societal and institutional context, encompasses the broader environment within which healthcare organizations operate. This may include legal and regulatory requirements, cultural and political conditions, governance arrangements, and public accountability mechanisms. Healthcare decisions may also have implications beyond the organization, including effects on access to services, public trust, and the distribution of benefits and burdens across different groups [16]. Relevant stakeholders may include patients and families, healthcare professionals, governing and regulatory bodies, payers, communities, and future service users, depending on the decision and healthcare context.

The fourth domain, temporal perspective, concerns how the implications of a decision may develop across different time horizons. Immediate benefits or burdens may differ from intermediate and longer-term consequences. At the same time, actions taken in the present may influence future service capacity, organizational sustainability, or the options available to subsequent decision-makers [1]. The EPDL therefore considers immediate, intermediate, and longer-term implications without assuming that any one time horizon should necessarily determine the evaluation of a decision.

Deliberation within the EPDL is represented as an iterative rather than strictly sequential pathway. For practical application, the pathway is organized into six broad steps. First, frame the decision and identify affected stakeholders. Second, consider the relevant ethical commitments and stakeholder perspectives. Third, reasonably available options are examined across four domains: ethical foundations, organizational realities, societal and institutional context, and temporal perspective. Fourth, the alternatives are compared and deliberated, with attention to their ethical implications, feasibility, trade-offs, and reasonably foreseeable consequences. Fifth, decision-makers identify the option judged to represent the Most Ethically Sustainable Decision (MESD), select a course of action, and proceed with implementation and monitoring. Sixth, the decision and its consequences are reviewed and reassessed as new information emerges, conditions change, risks become clearer, or stakeholder circumstances evolve. Reassessment may confirm the existing course of action, support its revision, or prompt renewed consideration of available alternatives.

Although presented as six steps for conceptual clarity, the pathway is not intended as a fixed algorithm. Decision-makers may move between steps as assumptions are reconsidered, new information becomes available, or relevant features of the decision landscape change. The pathway therefore provides a structure for deliberation and justification rather than a procedure that independently determines the MESD.

Ethical Elevation is proposed as a deliberative concept for comparing available options within the EPDL. It refers to the extent to which an option can be comparatively justified in relation to the ethical commitments relevant to the decision, while accounting for the contextual conditions that affect its feasibility and consequences. Ethical Elevation is not proposed as an objective numerical score, a universal moral ranking, or a claim of moral optimality. Rather, the concept makes comparative reasoning explicit by asking why one available option is considered more ethically defensible than another under the circumstances and how contextual conditions affect the feasibility and consequences of the alternatives being considered.

The MESD refers to the option that, following reasoned comparison of the available alternatives, is judged to be the most ethically defensible and practically sustainable within the circumstances of the decision. This judgment is informed by relevant ethical commitments and by organizational realities, societal and institutional context, and consequences across different time horizons that shape the available options and their implementation.

The term most is comparative rather than absolute: it refers to the options reasonably available within a defined context and does not imply an objectively optimal or morally perfect decision. The MESD may therefore change as relevant evidence, resources, risks, stakeholder circumstances, or feasible alternatives change.

The EPDL does not automatically generate or prescribe a decision. Rather, it provides a structure for deliberation in which decision-makers articulate the considerations regarded as relevant, explain how competing ethical claims and contextual considerations were evaluated, distinguish between constraints considered fixed and those potentially open to modification, and justify why one available option was preferred over the alternatives. The framework therefore emphasizes transparent, reasoned justification rather than treating the model itself as a source of ethical authority.

Conceptual Propositions

Proposition 11: Healthcare executive deliberation may be represented as navigation across the interacting domains of ethical foundations, organizational realities, societal and institutional context, and temporal perspective.

Proposition 12: Changes in evidence, resources, risks, stakeholder circumstances, or other contextual conditions may alter the feasibility, consequences, or comparative ethical defensibility of available options.

Proposition 13: Ethical Elevation is a deliberative concept for comparing available options and does not constitute an objective measure of moral quality.

Proposition 14: Identifying the MESD is context-dependent and may require reassessment when relevant features of the decision landscape change.

The Ethical Pragmatism Decision Landscape

Figure 1 presents the EPDL visually, showing the four domains and the six-step iterative deliberation pathway through which available courses of action may be examined, compared, implemented, and reassessed as relevant features of the decision landscape change.

**Figure 1:**
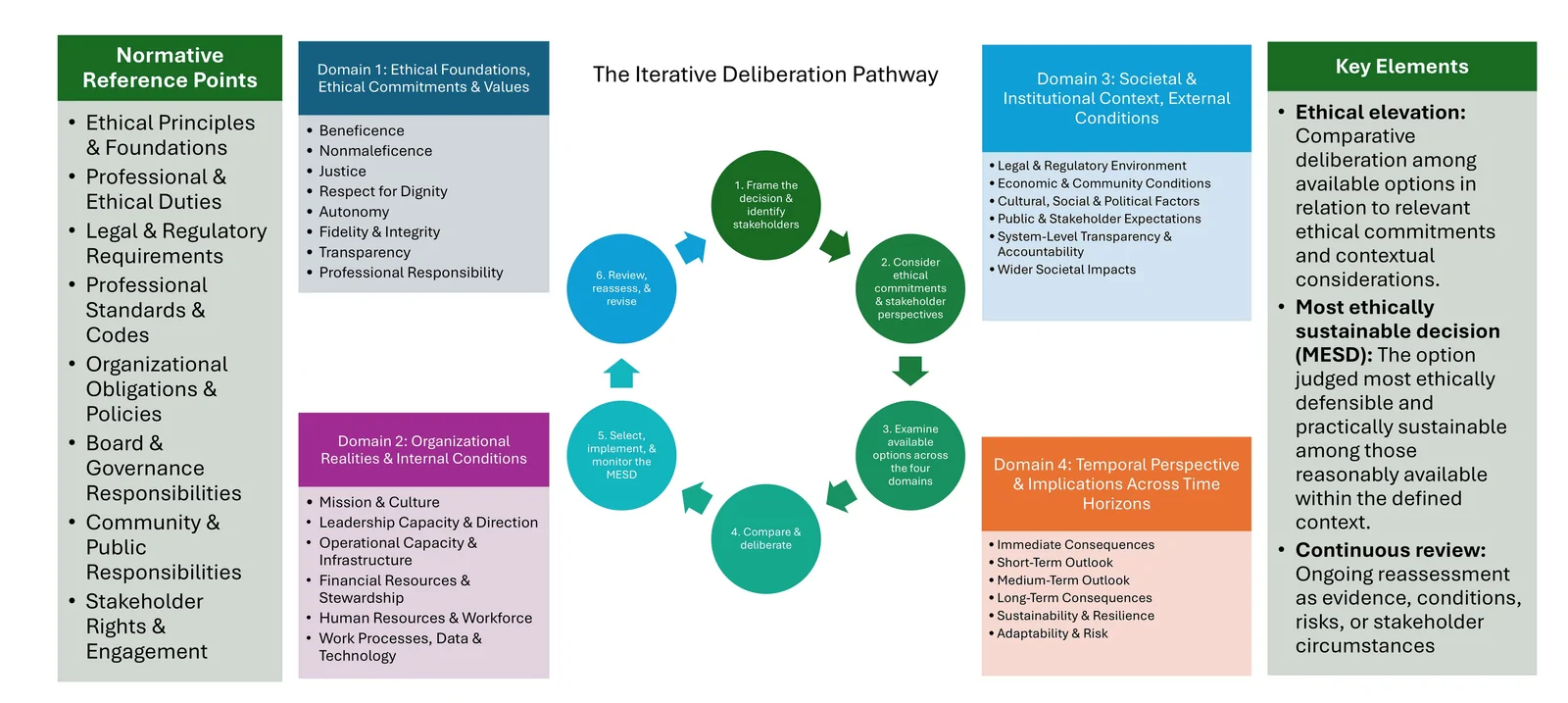
The Ethical Pragmatism Decision Landscape: An Iterative Framework for Identifying the Most Ethically Sustainable Decision (MESD) Source: Developed by the authors using Microsoft PowerPoint (Microsoft Corporation, Redmond, USA).

Practical applications

The EPDL may be applied through its iterative deliberation pathway, in which a decision is framed, relevant stakeholders and ethical commitments are identified, and reasonably available options are examined and compared across the four domains of ethical foundations, organizational realities, societal and institutional context, and temporal perspective. The process includes consideration of practical feasibility, reasonably foreseeable consequences, and whether relevant constraints are fixed or potentially modifiable, followed by selection, implementation, monitoring, and, where warranted, reassessment of the selected course of action. The examples below illustrate how the framework could structure deliberation in different healthcare-management contexts; they are not intended to demonstrate that its application improves decision quality or outcomes, which would require empirical investigation [8,16].

One illustrative application of the framework concerns resource allocation under conditions of scarcity. Decisions involving intensive care beds, operating theatre capacity, or other limited healthcare resources may involve multiple ethical considerations and be shaped by constraints in workforce, infrastructure, and service capacity. Through the EPDL, decision-makers can examine and compare reasonably available courses of action across ethical foundations, organizational realities, societal and institutional context, and temporal perspective. In doing so, decision-makers can consider the ethical commitments relevant to allocation, the conditions affecting feasibility and implementation, and the implications of alternative courses of action across different groups and time horizons. This process makes the grounds for the selected allocation more explicit, including the ethical reasoning and contextual conditions that informed the decision [6,8,16].

A second illustrative application concerns workforce planning. Decisions about staffing levels, skill mix, or workforce deployment may affect patient safety, workforce wellbeing, access to care, and the sustainable use of organizational resources. Through the EPDL, reasonably available options can be examined and compared across ethical foundations, organizational realities, societal and institutional context, and temporal perspective. This examination may include considerations such as patient safety and workforce wellbeing, workforce availability and financial capacity, wider societal and institutional implications, and consequences across different time horizons. The framework therefore provides a structure for considering workforce decisions beyond their immediate operational or financial implications without assuming that any single consideration determines the ethically preferable course of action [1,8].

A further illustrative application concerns emergency department overcrowding. Decisions about patient flow, staffing deployment, escalation measures, or changes to elective activity may affect patient safety and experience, workforce workload, access to services, and organizational capacity. Within the EPDL, reasonably available responses can be examined and compared across ethical foundations, organizational realities, societal and institutional context, and temporal perspective. Because demand, capacity, risks, and other relevant circumstances may change during an operational event, the framework allows iterative review and reassessment of both the available options and the judgment about which course of action represents the MESD under the prevailing circumstances [1,8].

Public health emergencies and disasters provide another illustrative application of the framework. Infectious disease outbreaks, mass casualty incidents, and similar events may require rapid decisions under uncertainty, in which obligations to individual patients and populations must be considered alongside workforce availability, organizational capacity, and evolving risks. Within the EPDL, reasonably available courses of action can be examined and compared across ethical foundations, organizational realities, societal and institutional context, and temporal perspective, recognizing that the circumstances affecting feasibility and foreseeable consequences may differ substantially from routine operating conditions. As those circumstances change, the judgment regarding which course of action represents the MESD may require reassessment. At the same time, relevant ethical and professional obligations continue to provide normative reference points [1,8,16].

The framework can also inform strategic and quality-improvement decisions. Investments in digital health, artificial intelligence, infrastructure, or new clinical services may raise ethical questions concerning benefit, equity, professional responsibility, and stewardship while also being shaped by financial resources, workforce capability, technological readiness, regulatory requirements, and implementation capacity. Within the EPDL, reasonably available strategic alternatives can be examined and compared across ethical foundations, organizational realities, societal and institutional context, and temporal perspective, including their implications for affected stakeholders and consequences across different time horizons. Similarly, quality-improvement initiatives can be examined not only in relation to intended operational gains but also in relation to potential effects on patient experience, professional practice, access, organizational capacity, and public accountability [1,8,12].

Across these illustrative examples, the EPDL serves a deliberately limited function: it provides a structure for making the grounds of a decision more explicit. It prompts decision-makers to identify the ethical commitments at stake, distinguish constraints treated as fixed from those potentially open to modification, consider how benefits and burdens may be distributed among affected groups, compare reasonably available alternatives, and examine consequences across different time horizons. Where relevant circumstances change, the framework also provides for reassessment of the selected course of action. In this sense, the EPDL structures deliberation and justification rather than determining the decision itself. Whether its application improves decision quality, perceived legitimacy, organizational outcomes, or other relevant outcomes remains a question for empirical investigation (Table 1).

**Table 1:** Illustrative Applications of the Ethical Pragmatism Decision Landscape (EPDL)

Healthcare challenge	Illustrative considerations across the EPDL	Illustrative role of the EPDL
Resource allocation under scarcity	Benefit, justice, and appropriate allocation; workforce and service capacity; legal and societal considerations; immediate and longer-term consequences.	Makes explicit the ethical justification, feasibility, contextual constraints, and consequences associated with available allocation options.
Workforce planning	Patient safety, workforce wellbeing, and access; workforce and financial capacity; operational requirements; short- and longer-term workforce consequences.	Examines staffing, skill-mix, and deployment options beyond their immediate operational or financial implications.
Emergency department overcrowding	Patient safety and experience; access; workforce and service capacity; patient flow; consequences as operational conditions change.	Supports iterative examination of available responses as demand, capacity, risks, and other conditions evolve.
Public health emergencies and disasters	Obligations toward patients and populations; workforce and organizational capacity; legal and institutional conditions; evolving risks and consequences.	Examines context-dependent alternatives under uncertainty and allows reassessment as emergency conditions change.
Strategic investment and service development	Benefit, equity, stewardship, and professional responsibility; financial and workforce capacity; technology and regulation; stakeholder and longer-term implications.	Examines strategic alternatives across the ethical, organizational, societal and institutional, and temporal perspectives.
Quality improvement	Patient safety and experience; professional practice; access; organizational capacity and accountability; immediate and longer-term consequences.	Examines intended improvements alongside potential ethical, professional, organizational, and wider implications.

Conceptual Propositions

Proposition 15: The EPDL may provide a conceptual structure for deliberating on healthcare executive decisions across operational, strategic, and emergency contexts.

Proposition 16: The relevance and relative significance of considerations within the EPDL may vary by decision context, and changes in relevant circumstances may warrant reassessing the available options and the selected course of action.

Proposition 17: Empirical research is required to determine whether and how the use of the EPDL is associated with decision quality, transparency, perceived legitimacy, or other relevant outcomes.

Implications for leadership, governance, and policy

The EPDL raises questions for leadership, governance, and policy concerning how healthcare organizations deliberate on and justify difficult decisions. One important implication is procedural: the framework directs attention to making relevant ethical commitments, organizational realities, societal and institutional considerations, and consequences across different time horizons visible within deliberation. Responsibility for examining these considerations may remain distributed across individuals, committees, and governance structures; the framework does not require combining these functions. Rather, it offers a common conceptual space in which their respective perspectives can inform the examination, comparison, and reassessment of available courses of action.

For healthcare leaders, the framework highlights a broader conception of executive accountability. Leadership decisions may be examined not only in relation to operational performance or financial outcomes but also in relation to their implications for patient welfare, workforce wellbeing, professional integrity, equity, public accountability, and organizational resilience. The relevance and relative significance of these considerations may differ by decision context. Within the EPDL, executive accountability therefore includes making explicit which considerations informed a decision, how competing claims were interpreted, and how the selected course of action was justified under the prevailing circumstances.

Within organizational governance, the EPDL could be used to organize the evidence and reasoning presented to boards, executive committees, ethics committees, quality councils, or other relevant governance bodies. The framework does not require merging these functions or their respective responsibilities. Rather, when a major decision has implications across several domains, the EPDL would bring relevant financial, clinical, legal, operational, and ethical perspectives into relation rather than examining each in isolation. A documented application of the EPDL could record the alternatives considered, affected stakeholders, relevant ethical commitments, contextual conditions and assumptions, reasonably foreseeable consequences, and the reasoning supporting the selected course of action.

A similar perspective may be relevant to health policy development. Policies directed toward goals such as safety, equity, accessibility, or quality are implemented within healthcare systems and organizations operating under particular resource, workforce, institutional, and operational conditions. The EPDL provides a structure for examining normative policy objectives alongside the conditions that shape their feasibility, implementation, and reasonably foreseeable consequences across different groups and time horizons. This perspective allows implementation constraints to be considered without treating feasibility alone as sufficient justification for modifying or limiting an ethically supported policy objective.

The framework may also have implications for leadership education. Ethical pragmatism offers a conceptual structure through which ethical reasoning, healthcare management, resource considerations, quality improvement, and systems perspectives can be examined within the same decision context. Case discussions, executive simulations, and interdisciplinary learning activities could use the EPDL to examine how participants identify relevant ethical commitments, assess contextual conditions, compare available alternatives, and justify decisions across the four domains. Whether such use contributes to the development of executive judgment or other leadership competencies would require empirical evaluation.

Finally, the framework may have implications for organizational culture. Applied within organizational dialogue, the EPDL could provide a common structure for making uncertainty, competing considerations, assumptions, and the reasoning behind difficult decisions more explicit. It may also support discussion of disagreement and examination of assumptions without presuming that every tension necessarily requires a trade-off. Its potential relationship with reflective dialogue, interdisciplinary collaboration, shared learning, and organizational accountability represents an area for future empirical investigation.

These implications remain conceptual proposals rather than demonstrated effects. The EPDL may offer a shared vocabulary for examining and discussing decisions across leadership, governance, policy, and education, but its practical value has yet to be established. Future evaluation should examine whether its domains and constructs are sufficiently clear for application, how its use influences deliberative processes, and whether it offers meaningful value beyond existing ethical, governance, and decision-making approaches (Table 2).

**Table 2:** Proposed Implications of the Ethical Pragmatism Decision Landscape (EPDL) and Questions for Future Evaluation

Area	Proposed implication of the EPDL	Question for future evaluation
Leadership	Makes explicit the ethical commitments, contextual considerations, and reasoning informing executive deliberation and decision-making.	Does use of the EPDL influence the transparency, quality, or perceived legitimacy of executive deliberation?
Governance	Provides a common conceptual structure for considering clinical, ethical, operational, financial, legal, and other relevant perspectives without requiring governance functions to be merged.	Can the EPDL be incorporated into existing governance processes, and does it add meaningful value beyond current approaches?
Health policy	Provides a structure for examining normative policy objectives alongside implementation conditions, affected groups, and consequences across different time horizons.	How does application of the EPDL influence policy deliberation under different institutional and resource conditions?
Leadership education	Offers a conceptual structure for examining ethical reasoning and contextual conditions within the same decision scenario.	Is the EPDL understandable and usable in case-based learning and executive simulation, and how does its use influence deliberative reasoning?
Organizational culture	Offers a common structure for making uncertainty, competing considerations, assumptions, and the reasoning behind difficult decisions more explicit.	What relationship, if any, exists between use of the EPDL and reflective dialogue, interdisciplinary collaboration, shared learning, or organizational accountability?

Conceptual Propositions

Proposition 18: Application of the EPDL within healthcare governance would emphasize documenting the considerations, assumptions, alternatives, and reasoning informing a decision, including subsequent reassessment where relevant, rather than prescribing a specific substantive outcome.

Proposition 19: The EPDL may be examined as an educational tool for structuring the analysis of ethically and contextually complex decisions in healthcare leadership education.

Proposition 20: Application of the EPDL to health policy could structure examination of normative objectives alongside implementation conditions, implications for affected groups, and consequences across different time horizons.

Future research

The Ethical Pragmatism framework requires further conceptual clarification and empirical examination. Initial qualitative research could explore how healthcare executives, clinical leaders, board members, and policymakers describe deliberation in ethically and contextually complex decisions, and examine the extent to which these accounts correspond with, challenge, or extend the domains, constructs, and iterative deliberation pathway proposed within the EPDL. Interviews, observations, and comparative case studies could identify considerations not adequately represented in the framework, examine how competing claims and contextual constraints are interpreted, and explore how decisions are reconsidered as circumstances change. Such research could contribute to refinement of the framework before more formal testing or measurement development is undertaken.

Further conceptual work should examine the relationship between the Ethical Pragmatism framework and related approaches to pragmatic moral reasoning. Richardson's constructive ethical pragmatism provides an important point of comparison because it attends to the articulation and revision of moral norms through practical reasoning [9, 10]. Donelson's account offers a different comparison by examining whether practical moral deliberation must depend on claims about moral truth or broader metaethical commitments [11].

Measurement research should proceed cautiously. Ethical Elevation and the MESD are currently proposed as deliberative concepts rather than validated empirical constructs. Before developing measurement instruments, research should examine whether these concepts can be clearly distinguished, defined with sufficient conceptual precision, and interpreted with reasonable consistency across decision-makers and contexts. If supported by such work, subsequent studies could investigate their relationships with deliberative processes and outcomes, including transparency, stakeholder participation, decision revision, perceived legitimacy, and relevant organizational outcomes. Such studies should distinguish association from causation and should not assume in advance that any future quantitative representation of these constructs would correspond directly to better decision-making.

Scenario-based research could examine how healthcare leaders interpret and apply the EPDL under varying conditions of uncertainty, resource scarcity, and time pressure, including how they move through or between the steps of the proposed iterative deliberation pathway. Comparative studies could explore whether the framework's domains and constructs remain applicable or require adaptation across different health systems, legal and institutional arrangements, and cultural contexts. The potential use of the EPDL in leadership education and digital decision-support systems represents another area for investigation. In digital applications, particular attention should be given to how decision-support tools might structure deliberation without treating Ethical Elevation or the MESD as objectively calculable outputs or displacing human responsibility for ethical judgment and justification.

Conceptual Propositions

Proposition 21: The extent to which the EPDL, including its proposed iterative deliberation pathway, adequately represents healthcare executive deliberation should be examined before claims are made about its practical effectiveness.

Proposition 22: Ethical Elevation and the MESD require further conceptual and empirical clarification before assumptions are made about whether, and how, they can be operationalized or measured.

Proposition 23: Potential applications of the EPDL in leadership education and digital decision-support systems require empirical evaluation of their usability, interpretability, and implications for human judgment and accountability.

## Conclusions

This paper develops a healthcare-management-specific account of ethical pragmatism for examining executive decision-making under conditions of ethical plurality, organizational complexity, uncertainty, and practical constraint. Rather than proposing an alternative normative ethical theory, the framework retains established ethical principles, professional obligations, legal requirements, and reasoned justification as normative reference points while recognizing that organizational, societal, institutional, and temporal conditions shape available courses of action. Its principal contribution, the EPDL, conceptualizes deliberation across four interacting domains (ethical foundations, organizational realities, societal and institutional context, and temporal perspective) and through a six-step iterative pathway of framing the decision, considering stakeholders and ethical commitments, examining options, selecting and implementing a course of action, and reviewing it as circumstances evolve. Ethical Elevation provides a comparative lens for considering the ethical defensibility of available options, while the MESD represents the option judged most ethically defensible and practically sustainable within a defined context; neither implies an objective moral ranking, automated calculation, or universally correct decision.

The EPDL therefore goes beyond a general call to reconcile ethics with practical realities by keeping normative commitments conceptually distinct from contextual conditions while examining how they interact in executive deliberation. Organizational feasibility influences what can be implemented and with what consequences, but feasibility alone cannot determine ethical acceptability. Ethical pragmatism thus offers a structured way of conceptualizing how healthcare leaders pursue ethical commitments within real-world constraints while preserving their normative force. Further conceptual and empirical research is needed to clarify the framework's relationship to existing ethical and governance approaches, examine its correspondence with executive decision-making in practice, and determine its value for understanding or structuring healthcare leadership deliberation.
